# Household Knowledge of Antimicrobials and Antimicrobial Resistance in the Wake of an Accredited Drug Dispensing Outlet (ADDO) Program Rollout in Tanzania

**DOI:** 10.1371/journal.pone.0163246

**Published:** 2016-09-29

**Authors:** Daudi Simba, Deodatus Kakoko, Innocent Semali, Anna Kessy, Martha Embrey

**Affiliations:** 1 Department of Community Health, School of Public Health and Social Sciences, Muhimbili University of Health and Allied Sciences, P.O. Box 65015, Dar-es-Salaam, Tanzania; 2 Department of Behavioural Sciences, School of Public Health and Social Sciences, Muhimbili University of Health and Allied Sciences, P.O. Box 65015, Dar-es-Salaam, Tanzania; 3 Department of Epidemiology and Biostatistics, School of Public Health and Social Sciences, Muhimbili University of Health and Allied Sciences, P.O. Box 65015, Dar-es-Salaam, Tanzania; 4 Management Sciences for Health, Arlington, VA, United States of America; Ross University School of Veterinary Medicine, SAINT KITTS AND NEVIS

## Abstract

**Introduction:**

Private sector drug shops are an important source of medicines in Tanzania. In 2003, the government introduced the accredited drug dispensing outlet (ADDO) program to improve access to good-quality medicines in rural and peri-urban areas that have frequent drug shortages in public health facilities and few or no registered pharmacies. However, increasing access may also contribute to antimicrobial resistance (AMR) due to the potential overuse and misuse of drugs.

**Methods:**

We conducted a cross-sectional household survey in four regions in mainland Tanzaniato characterize consumer care-seeking habits and medicines use and to determine the extent to which members of the community are knowledgeable about antimicrobials and AMR. Within the regions, we applied a multistage cluster sampling design, cascading from districts, wards, and villages to households. Multivariate logistic analysis was done to determine variables influencing knowledge of antimicrobials and AMR, while controlling for confounding factors. Variables included age, occupation, level of education, membership in an insurance scheme, and wealth status.

**Results and Discussion:**

We revealed that communities in four Tanzanian regions have low levels of knowledge of the concepts of antimicrobials and their use and AMR. Level of public understanding rose with wealth status and education. Only one-third of 1,200 respondents (33.6%) had ever heard of a medicine called an antimicrobial, and 5–15% could name at least one antimicrobial spontaneously. Some thought other medicines, such as paracetamol were antimicrobial (7.5%). People were equally likely to agree that pneumonia should be treated with an antimicrobial (21.4%) as well as common cold (28.4%). Understanding of AMR risks was better, particularly related to HIV and AIDS (32.2%) and malaria (38.6%)—most likely due to information campaigns focused on those two diseases. The level of knowledge decreased the further away respondents lived from an ADDO (p = 0.0001) and where ADDO density was lower (p = 0.001), which supports the use of ADDO dispensers as sources of community information and change agents for more appropriate medicine use.

**Conclusion:**

Lack of knowledge about antimicrobials and AMR in Tanzanian communities needs to be addressed through multi-pronged strategies that focus on prescribers and the public—especially those who are poorer and less educated.

## Introduction

Efforts to control infectious diseases are challenged by the emergence of antimicrobial resistance (AMR) that has been increasing globally, with the primary contributors being overuse and misuse of antimicrobials[1,2]. AMR also places financial burden on countries with weak economies due to the higher costs of alternative antimicrobials [3].In addition, alternative treatments are often more toxic and associated with more and serious adverse events [4].

In the last decades, the World Health Organization (WHO) has spearheaded global efforts to control the emergence of AMR by advocating for using medicines appropriately. Rational medicine use relies on prescribers and patients whose attitudes toward antimicrobial use drive their behaviors. While prescribers play a major role in ensuring the right medicine is given for the right disease in the right dose and duration, patients also have to adhere to the prescriptions.

The privatesector is acknowledged as an important source of medicines in many countries, including Tanzania [5,6].In an effort to improve the quality of pharmaceutical products and services in the private sector, in 2003, Tanzania introduced the accredited drug dispensing outlet (ADDO) program, which combined owner and dispenser training, government accreditation based on standards, business incentives, and local regulatory enforcement, with efforts to increase consumer demand for quality products and services. The program was begun with the goal of improving access to good-quality medicines in rural and peri-urban areas that have frequent drug shortages in public health facilities and few or no registered pharmacies.

To date, the program has been rolled out throughout the country with more than9,000 ADDOs established and 19,000 dispensers trained [7]. Dispenser training covers topics such as diagnosis and management of common conditions, patient counseling, and referral to a health care facility. After meeting the accreditation standards, dispensers are allowed to dispense, some prescription-only medicines, including selected antimicrobials that are on a list approved and issued by the Ministry of Health and Social Welfare. While this flexibility is aimed at improving access to antimicrobials in underserved areas, it may also contribute to AMR due to the potential overuse and misuse of drugs, which are beyond the control of regulatory enforcement efforts [8].

This household survey was part of a multi-method assessment to explore the different components that contribute to antimicrobial access and use in Tanzania with a focus on ADDOs. The purpose of the household survey was to characterize consumer care-seeking habits and medicines use and to determine the extent to which members of the community are knowledgeable about antimicrobials and AMR. Other parts of the study used interviews with patients leaving health facilities to uncover health facility prescribing and dispensing practices, a mystery shopper exercise to determine ADDO dispensing practices, and audits of ADDOs and health facilities to evaluate the availability and prices of antimicrobials. Results from other parts of the study have been published elsewhere [9,10].

## Methods

### Study Setting

Tanzania’s mainland has 947,300 square kilometers with 43,625,354 inhabitants located in 25 regions with populations ranging from 1.1 to 3.5 million people. A region consists of four to eight districts, with the population in each district ranging from 150,000 to 450,000 in rural areas and up to one million in some urban areas[11]. The districts are divided into wards that are further sub-divided into villages (rural areas) or streets (urban settings).

The health system is a pyramid with a narrow apex comprised of one national and three zonal consultant hospitals and a broad base made up of 5,657 dispensaries providing care at village level,653 health centers at the ward level, and one hospital in each district. Services are decentralized, and thus, district councils are responsible for overseeing their respective dispensaries, health centers, and hospital. About half of the health facilities are owned by the government, while the remaining half are run by nongovernmental, private for-profit, or religious organizations. Some wards have many ADDOs (high density) while others have few (low density) or none.

### Study Design and Sampling Procedure

We conducted this cross-sectional study in four regions of Tanzania’s mainland: Mbeya, Morogoro, Tanga, and Singida. Selection of the four regions was purposive, based on geographical location, wealth status, and the length of time that the ADDO program had been implemented. Morogoro represented the eastern regions and had more than five years of experience in implementing ADDOs. Tanga region represented the northern regions, and the program had been operational for about two years. Mbeya region was selected to represent the southern highlands and had had ADDOs for about three years. It also has a relatively high wealth status compared to the other selected regions. Singida represented the central regions, and at the time of the study, had been implementing the ADDO program for less than one year; it has a relatively low wealth status compared to the other selected regions. We aimed to reach a sample of 1,200 households. Calculation of the sample size assumed a 95% confidence interval (CI), a non-response rate of 10%, and a design effect of 2.0.

**Fig 1:**
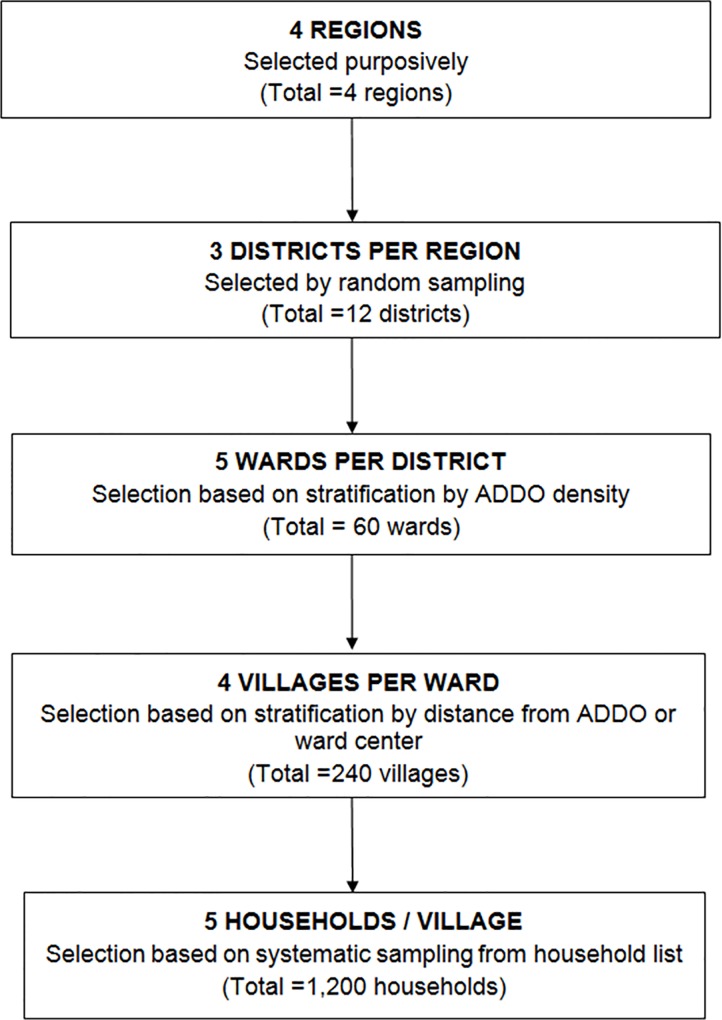
Sampling design.

Within the regions, we applied a multistage cluster sampling design, cascading from districts, wards, and villages to households as illustrated in Fig 1.

### Data Collection Procedures

We recruited a team of 20 research assistants and trained them on the research objectives, field work procedures, and the use of electronic tablets to capture data. The team participated in a two-day pilot study in Coast region to consolidate field experiences and identify and respond to logistical challenges with conducting the actual survey.

A village guide introduced the data collector to a household member, and together they identified an appropriate respondent, defined as a member of the household who was most informed about the health status and access to health care of the other members. The respondent was interviewed after signing an informed consent form.

We adapted to local context the WHO household survey on medicine access and use[12] and an AMR population-based questionnaire used in Demographic and Health Surveys[13] (S1. Questionnaire). The resulting questionnaire contained 40 questions related to general information about the household, access to health care services, and knowledge of antimicrobials and AMR. We used the questionnaire to establish knowledge on four main study questions: First, respondents were asked if they were aware of antibiotics. Those who responded affirmatively were asked to mention the names of some antibiotics they knew. Responses were recorded as “spontaneous” (for those who mentioned names without probing);“after probing” using a list of medicines that included both antibiotics and non-antibiotics; and “don’t know.” Respondents were asked to mention illnesses in which antibiotics were useful. Then, the interviewers defined what AMR means as follows, “Some medicines that used to work in the past for fighting infections are no longer working. This problem is called drug resistance.”The interviewers asked the respondents if they had ever heard of the problem. The interviewers mentioned a list of acute illnesses to test respondents’ knowledge ofillnesses in which serious treatment resistance has been reported. Finally, the interviewers further probed on knowledge of factors that contribute to resistance by reading to them a number of correct and incorrect options.

### Data Management and Analyses

The electronic tablet software used for data collection had been formatted to check inconsistencies, skipping patterns, and outliers. Daily, the data collection supervisor copied data from research assistants and checked for errors and inconsistencies prior to uploading it to a central server via the Internet. Data was then transferred to STATA version 10.0 (Stata Corp., College Station, TX, USA) for analysis (S1 and S2 Data).

Analyses involved the calculation of frequencies and cross-tabulations to identify associations. We measured strength of association using chi-square tests and Fisher exact tests (for tables with cells that had a value of less than 5);p-values lower than 0.05 were considered significant. Logistic regression was used in multivariate analyses to calculate crude odds ratios (OR), adjusted OR (AOR) in multivariate analysis, and the respective 95% CI.

Each of the four study questions had a range of four to seven correct responses. A score was assigned for each correct response as follows:

Question 1: Please mention the names of some antibiotics that you know. This question had seven correct responses. A score was assigned if a response was given spontaneously.

Question 2: For which of the following illnesses or conditions are antibiotics useful? (Interviewer mentions and respondents agree or disagree). The question had seven correct responses. Although the question was specifically about antibiotics, respondents could include antimicrobials that are not antibiotics (since differentiating the two requires expert knowledge) [14]. For this reason, during analyses, we treated all antimicrobials as correct responses.

Question 3: Can you tell me which of the following illnesses have more serious resistance problems? (Interviewer mentions and respondents agree or disagree). This had four correct responses.

Question 4: Can you tell me whether each of the following can contribute to causing resistance problems? (Interviewer mentions and respondents agree or disagree). This had six correct responses.

We measured knowledge of antimicrobials by adding the sum score of correct responses from the first question; scores of 0 indicated low knowledge, while scores of one and above indicated high knowledge. We measured knowledge of AMR by adding the sum score of correct responses from questions 3 and 4. Respondents scoring 0 were labeled as having no knowledge; those scoring from 1 to 8 were labeled with some knowledge; and those with scores of 9 and 10 had good knowledge.

We measured wealth status using asset ownership based on a list adopted from the Demographic Health Survey [15].Principal component analysis was used to create a wealth index that categorized households into wealth quartiles. Items owned or not owned by less than 10% were excluded. Excluded items were vehicles, tractors, power-tillers, electric kettles, microwaves, and toilets.

Multivariate logistic analysis was done to determine variables influencing knowledge ofantimicrobials and AMR, while controlling for confounding factors at a level of significance of p-value <0.1. We used a step-wise approach to enter variables into the model until attaining the highest regression coefficient. The variables were age, occupation, level of education, membership in an insurance scheme, and wealth status.

### Ethical Clearance/Considerations

The study was granted ethical clearance from Muhimbili University of Health and Allied Sciences Institutional Review Board and the National Institute for Medical Research. We presented letters of introduction to regional and district leaders and delivered similar letters from the districts to ward and village leaders whom we briefed on the project objectives. The data collectors asked the selected household respondents to give signed consent, and only those who consented were interviewed. If the respondent declined to be interviewed, the research assistant moved to the next household.

## Results

In this study, we assessed respondents’ awareness and knowledge about antimicrobials and antimicrobial resistance. About one-third of the respondents (33.7%) reported being awareness on antimicrobials (Table 1). Awareness of antimicrobials decrease significantly with ADDO density, from 41.8% in the high-density areas to 19.7% in the areas without ADDOs (p = 0.001).Although awareness of antimicrobials did not vary significantly across regions, respondents residing closer to an ADDO (<5kms) were more likely to be aware of antimicrobials compared to their counterparts living farther away from an ADDO (38.9% and 20.7%, respectively, p = 0.0001).

**Table 1:** Awareness of antimicrobials as a type of medicine by region, ADDO density, and distance from ADDO.

Factor	N	Not aware (%) (n = 796)	Aware (%)(n = 404)	P-value
**Region**				
Mbeya	300	68.3	31.7	0.285
Morogoro	300	62.7	37.3	
Singida	300	69.3	30.7	
Tanga	300	65.0	35.0	
**ADDO density**				
High	569	58.2	41.8	0.001
Low	453	71.1	28.9	
None	178	80.3	19.7	
**Distance from ADDO**				
<5kms	857	61.1	38.9	0.0001
>5kms	343	79.3	20.7	

When respondents who had heard of antimicrobialswere asked to mention any antimicrobialthey knew,the proportion mentioning a correct antimicrobial spontaneously was quite low, ranging from 4.9% for sulfadoxine-pyrimethamine to 15.2% for amoxicillin (Table 2 Question 1). After probing using a list of medicines, more respondents agreed that most of the medicines were antimicrobials, even if they were not; for example, 20.9% thought mist expectorant sedative was an antimicrobial.

**Table 2:** Responses to Questions about antimicrobials and antimicrobial resistance**.

**Question 1: Please mention the names of some antibiotics that you know**	**Mentioned spontaneously**	
	**N**	**(%)**
Amoxicillin*	182	15.2
Tetracycline*	110	9.2
Cotrimoxazole*	103	8.6
Paracetamol	90	7.5
Metronidazole*	81	6.8
Artemether-lumefantrine*	80	6.7
Penicillin*	71	5.9
Sulfadoxine-pyrimethamine*	59	4.9
Mist expectorant sedative	34	2.8
**Question 2: For which of the following illnesses or conditions are antibiotics useful? (Interviewer mentions and respondents agree or disagree)**	**Responded yes**	
	**N**	**(%)**
Cough, cold, and runny nose	341	28.4
Malaria*	318	26.5
Diarrhea with bloody stool*	273	22.8
Diarrhea with watery stool	275	22.9
Pneumonia*	257	21.4
Sexually transmitted infection*	217	18.1
Ear discharging pus, earache*	186	15.5
Tuberculosis*	136	11.3
HIV/AIDS*	68	5.7
**Question 3: Can you tell me which of the following illnesses have more serious resistance problems? (Interviewer mentions and respondents agree or disagree)**	**Responded yes**	
	**N**	**(%)**
Malaria*	463	38.6
HIV/AIDS*	386	32.2
Tuberculosis*	298	24.8
Sexually transmitted infection*	271	22.6
Cough, cold, and runny nose	268	22.3
Pneumonia	180	15.0
Ear discharging pus, earache	178	14.8
Diarrhea with bloody stool	135	11.3
Diarrhea with watery stool	126	10.5
**Question 4: Can you tell me whether each of the following can contribute to causing resistance problems? (Interviewer mentions and respondents agree or disagree)**	**Responded yes**	
	**N**	**(%)**
Taking medicines that have been kept at home for a long time*	550	45.8
Stopping taking medicine before finishing the treatment duration*	537	44.8
Using medicines that are poorly manufactured*	535	44.6
Taking less of the medicine at one dose than is recommended*	529	44.1
Skipping doses of the medicine*	522	43.5
Taking more of the medicine at one dose than is recommended	501	41.8
Using medicines that were prescribed for someone else*	501	41.8
Taking different medicines at the same time that interfere with each other	482	40.2
Taking the same medicine too frequently	449	37.4
Taking medicines without eating any food	445	37.1

*Correct response

**We considered any antimicrobial to be a correct answer

We further explored antimicrobialsknowledge by using a list of conditions (Table 2 Question 2) to prompt respondents to agree or disagree with whether or not the illness is treatable with antimicrobials. Malaria (26.5%) and bloody diarrhea (22.8%) were the most common illnesses that respondents knew to be treatable with antimicrobials. However, an equally similar proportion of respondents erroneously agreed that antimicrobials can be used to treat watery diarrhea (22.9%) and cough and cold (28.4%).

To explore respondents’ awareness of AMR in the community, we assessed their knowledge of resistance to standard treatments by prompting them using a list of specific diseases. While only one-quarter of the respondents correctly agreed that resistance to antimicrobial agents exists with sexually transmitted infections (22.6%) and tuberculosis (24.8%),about one-third agreed on AMR related to HIV/AIDS (32.2%) and malaria (38.6%) (Table 2, Question 3).

Nearly one-half of the respondents thought that stopping a medicine before finishing the prescribed treatment duration (44.8%)and also taking less of the medicine at one dose than prescribed(44.1%) contribute to AMR(Table 2). The high response rate might not actually indicate good knowledge of factors contributing to AMR because similar proportions affirmed unlikely contributors, such as taking more of the medicine at one dose (41.8%) and taking medicine without eating (37.1%).

Table 3 shows that the level of knowledge on antimicrobials was associated with the region the respondent came from (p = 0.0001), ADDO density in the respective district (p = 0.0001) and distance from household to an ADDO (p = 0.0001).A similar pattern was observed when analysis was done with knowledge on AMR.

**Table 3:** Overall levels of knowledge of antimicrobials and AMR by region, ADDO density, and distance from an ADDO*.

	**Level of knowledge on antimicrobials** (%)			**p-value**
**Region**	**None**	**Low**	**High**	
Mbeya	68.7	18.3	13.0	0.0001
Morogoro	64.3	27.7	8.0	
Singida	70.3	24.7	5.0	
Tanga	66.7	39.7	3.7	
ADDO density				
High	58.7	33.6	7.7	0.0001
Low	72.9	19.4	7.7	
None	82.0	12.4	5.6	
**Distance from ADDO**				
<5km	62.3	28.8	8.9	0.0001
≥5km	80.5	15.7	3.8	
	**Level of knowledge on AMR (%)**			**p-value**
**Region**	**None**	**Low**	**High**	
Mbeya	50.3	31.3	18.3	0.005
Morogoro	51.0	26.3	22.7	
Singida	46.7	27.3	26.0	
Tanga	57.7	29.0	13.3	
**ADDO density**				
High	46.4	31.8	21.8	0.01
Low	54.3	25.8	19.9	
None	60.1	24.7	15.2	
**Distance from ADDO**				
<5km	48.2	29.4	22.4	0.001
≥5km	59.5	26.2	14.3	

*Levels of knowledge based on average scores on responses to questions in Table 2.

We performed logistic analysis to establish the influence of the following factors on level of knowledge:age, occupation, level of education, membership in an insurance scheme, and wealth status. Table 4 shows that respondents in wealthier households (2^nd^–4^th^quartile) were five times more likely to have a higher knowledge of antimicrobials compared to those from poorer households (1^st^quartile)(AOR = 3.21;CI = 3.21–7.54).Respondents who completed primary education were three times more likely to have more knowledge than those with no or incomplete primary education(AOR = 3.23; CI = 2.49–4.18).We observed similar findings related to AMR knowledge, although the adjusted odds ratios were lower (AOR = 1.97; CI = 1.48–2.61and AOR = 2.16; CI = 1.74–2.70, respectively).

**Table 4:** Logistic analysis of factors influencing respondents’ level of knowledge of antimicrobials and AMR.

Factors related to antimicrobials knowledge	OR	95% CI	AOR	95% CI
Wealth status	7.17	4.76–10.81	4.92	3.21–7.54
Education	3.75	2.94–4.79	3.23	2.49–4.18
Occupation	1.46	1.34–1.59	1.32	1.20–1.45
Age	1.21	1.02–1.44	1.57	1.28–1.93
Membership in insurance scheme	1.00	0.73–1.36	1.01	0.75–1.44
**Factors related to AMR knowledge**				
Wealth status	2.43	1.86–3.18	1.97	1.48–2.61
Education	2.22	1.80–2.72	2.16	1.74–2.70
Occupation	1.13	1.04–1.23	1.03	0.95–1.13
Age	1.16	0.99–1.36	1.32	1.11–1.57
Membership in insurance scheme	1.07	0.80–1.43	1.06	0.79–1.44

## Discussion

We revealed that communities in four Tanzanian regions had low levels of knowledge of the concepts of antimicrobials and their use and AMR. However, studies in both developed and developing countries have found similar results, with the level of public understanding generally rising with socioeconomic status and education [16–18], which we also found. In Tanzania, a person who is economically better off is more likely to own a radio or a television and have more access to reading materials related to antimicrobials and AMR; while half of Tanzanians own a radio (55%) and only 16% own a television set [19].

In our study, few household respondents were able to spontaneously name antimicrobial correctly, and many thought that other medicines such as paracetamol and mist expectorant sedative were antimicrobials. The term “antibiotic” is an alien concept to most members of the local community, shown by the lack of a similar word in Kiswahili; the word “antibayotiki” has been adapted from English. People tend to comprehend modern or Western concept of diseases and medicines through their own local understanding, known as a local explanatory model [20,21],by mixing local/traditional and modern medical concepts [1].This phenomenon might explain some of the respondents’ misconceptions about, where some thought that mist expectorant sedative is an antimicrobial and that antimicrobials can be used to treat watery diarrhea and cold and coughs. The misconception might also result from people’s inability to discern the difference between symptoms and disease, for example, when they observe doctors prescribing amoxicillin and mist expectorant sedative interchangeably or together to “treat” a cough.

Community understanding of the concept of AMR was better. A quarter of the respondents knew of serious AMR issues related to tuberculosis and sexually transmitted infections; however, one-third knew about serious resistance against antiretroviral drugs, perhaps because of large investments to raise awareness about HIV/AIDS. In addition, the Kiswahili term “usugu” can be translated to mean resistance, but can also mean chronic disease. Because HIV/AIDS is a chronic disease, respondents could translate “usugu” as that instead of as resistance to drugs. The largest proportion of respondents reported awareness of resistance against malaria parasites, which might be explained by the previous decade’ strong public campaigns related to the resistance of chloroquine and sulfadoxine-pyrimethamine and the shift to the newly recommended artemisinin-based combination therapy.

We found that the level of knowledge of antimicrobials and AMR decreased if respondents lived further from an ADDO and where ADDO density was lower. This may support the important role that ADDO providers and the program in general play in communicating the correct information about antimicrobials and AMR to the community. Tanzanian drug sellers reportedly have a better understanding of antimicrobials and AMR compared to the general population [22,23],which may be attributed to the ADDO training that includes, among other things, rational medicines use and counselling [24]. In addition, most ADDO dispensers are nurse assistants with one year of health training, which could have increased their medicine knowledge.

We expected that regions with a longer-running ADDO program would have a higher proportion of household respondents with the correct knowledge ofantimicrobialsand AMR. However, our findings showed that although Morogoro region started much earlier it did not excel over the other three regions. In fact, after the initial ADDO training, there has not been any continuing education for dispensers. Furthermore, anecdotal evidence indicates that the accreditation that the ADDO dispensers receive motivates them to seek better-paying jobs in urban areas. Thus, replacement of trained with untrained providers may have compromised the level of knowledge in regions with long-standing programs. Recently, however, training institutions located in each of Tanzania’s seven zones have begun offering regular ADDO dispenser training, which will help ensure a consistent supply of trained people [7].

## Methodological Considerations

In this study, we did not weight the data; hence, comparisons between regions require caution given the large population size differences. In addition, the interpretation of the term “resistance” to mean disease chronicity might have led to over-reporting of AMR knowledge for conditions such as HIV/AIDS. Because respondents were likely to include all types of antimicrobials while responding questions related to antibiotics, we considered all antimicrobials and diseases treated by antimicrobial drugs as correct responses during analysis. We made few changes to the original questions; however, the translation of concepts into Kiswahili may have affected the validity. Therefore, comparison with other countries needs to be done with caution given existing differences in local context.

## Conclusions

Tanzanians’ limited knowledge ofantimicrobialsand AMR is likely to have negative implications for efforts to reduce the emergence of AMR, because people with a poor understanding of the related concepts are unlikely to adopt the desired behavior changes.

Strategies that lean too heavily on professional education are equally unlikely to result in large-scale or long-lasting improvement [1].Therefore, multi-pronged strategies are needed to improve the level of information about antimicrobials, the effects of irrational use of medicines, and the dangers of AMR. For example, in addition to incorporating medicine use topics in health care provider curricula, public advocacy campaigns can encompass a variety of interventions including focusing on school-aged children[25] or patients waiting to be attended, taking advantage of the long waiting times at health facilities, and using of mass media to raise awareness in the community. Since low-socioeconomic status households and people with less formal education are more likely to have poor knowledge of AMR, the education and communication strategy should target rural remote areas and urban slums where the majority of the poor and the undereducated reside.In addition, ADDO dispensers should be used as change agents because they serve many community members due to the large number and geographic spread of shops and the frequent drug stock-outs in public facilities.

## Supporting Information

S1 Data PLoS One Summary.dta. (DTA)

S2 Data PLoS One Summary.do. (DO)

S1 Questionnaire Household Survey on Medicines Use and Knowledge of AMR. (DOCX)
