# Supplementary material for: Household Knowledge of Antimicrobials and Antimicrobial Resistance in the Wake of an Accredited Drug Dispensing Outlet (ADDO) Program Rollout in Tanzania
Source: PLoS One. 2016 Sep 29;11(9):e0163246. doi: 10.1371/journal.pone.0163246 (PMC5042404; doi:10.1371/journal.pone.0163246)
Supplement: S1 Questionnaire — (DOCX) [file pone.0163246.s003.docx]

| **0-1Region ____________** | | | **0-2Enumerator____________** | | | | **0-3Date_ _/_ _/_ _ _ _***(dd/mm/yyyy)* | | |
| --- | --- | --- | --- | --- | --- | --- | --- | --- | --- |
| **0-4District­________** | **0-5 Ward__________** | | **0-6 ADDO Density:** | | | \| 1High \| \| --- \| | | \| 2Low \| \| --- \| | \| 3None \| \| --- \| |
| **0-7 Distance to closest ADDO:** | | 1**<5 km** | | 2**>5 km** |  | | | | |

**The survey should not be completed if the main health care decision makeris not available,, or appropriate substitute is absent.An appointment should be scheduled to interview him/her onthe same day or the following day.**

*The person who makes decisions about health care in this household, or appropriate substitute, is available to answer:*

**1 Yes 🡪 if Yes, Continue 2 No 🡪 if No, Stop here.**

**Part One: Household Roster**

1. Please give the name, sex and age, relationship to head of household, education, occupation, and marital status of each household member who lives here, **STARTING WITH THE RESPONDENT.***Write one person per row starting with the respondent and use codes provided in each column to complete each row.*

|  | **A** | **B** | **C** | | **D** | **E** | **F** | **G** |
| --- | --- | --- | --- | --- | --- | --- | --- | --- |
|  | **Name** | **Sex** | **Age** | | **Relationship to head** | **Highest Level of Education** | **Occupation** | **Marital status** |
| **R**  **O**  **S**  **T**  **E**  **R**  **N**  **U**  **M**  **B**  **E**  **R** | *Write a name that identifies each member (initials, first name, nick name, or complete name…). Always write the respondent info on row one* | *1=Male*  *2=Female* | *Write number of years in the Years column.*  *Use the Month column only if less than 1 year old.*  *Years Months* | | *1=Head*  *2=Spouse*  *3=Child*  *4=Grandchild*  *5 =Parent*  *6 =Sibling*  *7=Nephew/niece*  *8=Other family member*  *9=House helper*  *10= Friend*  *99=Other (specify)* | *1=No formal schooling*  *2=Some primary*  *3=Completed primary*  *4=Completed secondary*  *5=Completed high school or equivalent*  *6=Completed college/university*  *7=Completed post-graduate* | *1=Farmer/fisherman*  *2=Teacher*  *3=Artisan*  *4=Office worker*  *5=Civil Servant*  *6=Agric/fish labor*  *7=Non-agric labor*  *8=Health worker*  *9=Self-employed/own business*  *10=Student/pupil*  *11=Unemployed*  *12=Not in labor force/retired*  *99=Other (specify)* | *1=Married*  *2=Consensual union*  *3=Divorced*  *4=Separated*  *5=Widowed*  *6=Never married*  *7=Non Applicable* |
| **01** |  |  | **\|__\|\|__\|\|__\|** | **\|__\|\|__\|** |  |  |  |  |
| **02** |  |  | **\|__\|\|__\|\|__\|** | **\|__\|\|__\|** |  |  |  |  |
| **03** |  |  | **\|__\|\|__\|\|__\|** | **\|__\|\|__\|** |  |  |  |  |
| **04** |  |  | **\|__\|\|__\|\|__\|** | **\|__\|\|__\|** |  |  |  |  |
| **05** |  |  | **\|__\|\|__\|\|__\|** | **\|__\|\|__\|** |  |  |  |  |
| **06** |  |  | **\|__\|\|__\|\|__\|** | **\|__\|\|__\|** |  |  |  |  |
| **07** |  |  | **\|__\|\|__\|\|__\|** | **\|__\|\|__\|** |  |  |  |  |
| **08** |  |  | **\|__\|\|__\|\|__\|** | **\|__\|\|__\|** |  |  |  |  |
| **09** |  |  | **\|__\|\|__\|\|__\|** | **\|__\|\|__\|** |  |  |  |  |
| **10** |  |  | **\|__\|\|__\|\|__\|** | **\|__\|\|__\|** |  |  |  |  |
| **11** |  |  | **\|__\|\|__\|\|__\|** | **\|__\|\|__\|** |  |  |  |  |
| **12** |  |  | **\|__\|\|__\|\|__\|** | **\|__\|\|__\|** |  |  |  |  |
| **13** |  |  | **\|__\|\|__\|\|__\|** | **\|__\|\|__\|** |  |  |  |  |
| **14** |  |  | **\|__\|\|__\|\|__\|** | **\|__\|\|__\|** |  |  |  |  |
| **15** |  |  | **\|__\|\|__\|\|__\|** | **\|__\|\|__\|** |  |  |  |  |
| **16** |  |  | **\|__\|\|__\|\|__\|** | **\|__\|\|__\|** |  |  |  |  |
| **17** |  |  | **\|__\|\|__\|\|__\|** | **\|__\|\|__\|** |  |  |  |  |
| **18** |  |  | **\|__\|\|__\|\|__\|** | **\|__\|\|__\|** |  |  |  |  |
| **19** |  |  | **\|__\|\|__\|\|__\|** | **\|__\|\|__\|** |  |  |  |  |
| **20** |  |  | **\|__\|\|__\|\|__\|** | **\|__\|\|__\|** |  |  |  |  |

**Part Two: Health Services Access & Illnesses**

1. How much time does it take you to walk to the following health care facilities or providers that are closest to your household? *Read responses and tick one box for each one of the categories.*

|  | **< 15 min** | **15 min to 1 hr** | **> 1 hr** |  |  | **< 15 min** | **15 min to 1 hr** | **> 1 hr** |
| --- | --- | --- | --- | --- | --- | --- | --- | --- |
|  |  |  |  |  |  |  |  |  |
| Public hospital | 1 | 2 | 3 |  | ADDO | 1 | 2 | 3 |
| 1. NGO or mission hospital | 1 | 2 | 3 |  | 1. Private pharmacy | 1 | 2 | 3 |
| Public health center or dispensary | 1 | 2 | 3 |  | Drugstore (DLDB) | 1 | 2 | 3 |
| 1. Private hospital or dispensary | 1 | 2 | 3 |  | Ordinary shop | 1 | 2 | 3 |

1. Is your household currently enrolled in any health insurance scheme? **1 Yes 2 No if No skip to 2-4**
2. If yes, mention which

| 1CHF | 2NHIF | 3Others (specify)…………………..……………. |
| --- | --- | --- |

1. An acute illness is a condition that appears suddenly: the person did not have it immediately before becoming ill. Has anyone in this household been ill in the past two weeks with an acute illness?

1 Yes 0 No **🡪 If No, Skip to Question 2-6**

1. Can you give the name of each person in the household who had an acute illness over the past two weeks? **START WITH THE PERSON WHO GOT SICK MOST RECENTLY**. *Transcribe name and roster number from the household roster.* Later on, I will ask you a series of questions about each of these people.

| **Name (as in roster)** | **Roster number** | **Acute illness module completed** | |
| --- | --- | --- | --- |
|  |  | **Yes** | **No** |
|  | **\|__\|\|__\|** | 1 | 0 |
|  | **\|__\|\|__\|** | 1 | 0 |
|  | **\|__\|\|__\|** | 1 | 0 |
|  | **\|__\|\|__\|** | 1 | 0 |
|  | **\|__\|\|__\|** | 1 | 0 |
|  | **\|__\|\|__\|** | 1 | 0 |
|  | **\|__\|\|__\|** | 1 | 0 |
|  | **\|__\|\|__\|** | 1 | 0 |
|  | **\|__\|\|__\|** | 1 | 0 |

1. A chronic disease is an illness that will not go away or takes a long time to go away, even when treated Has anyone in this household **ever been told** by a doctor or other health care providers that he/she has a chronic disease?.

1 Yes 0 No **🡪 If No, Skip to Question 3-1**

1. Can you give me the name of each person in the household with a chronic disease?*Transcribe name and roster number from the household roster.* Later on, I will ask you a series of questions about each of these people and their disease.

| **Name (as in roster)** | **Roster number** | **Chronic disease module completed** | |
| --- | --- | --- | --- |
|  |  | **Yes** | **No** |
|  | **\|__\|\|__\|** | 1 | 0 |
|  | **\|__\|\|__\|** | 1 | 0 |
|  | **\|__\|\|__\|** | 1 | 0 |
|  | **\|__\|\|__\|** | 1 | 0 |
|  | **\|__\|\|__\|** | 1 | 0 |

*First, complete one acute module (Part Four) for each person mentioned with an acute illnessin Questions 2-5 above. After completing each person, check ‘Yes’ under “Acute illness module completed” in the corresponding row.*

*After all acute illness modules have been completed, fill in one chronic module(Part Five) for each person with a chronic disease mentioned in Question 2-7 above. After completing information about each person, check ‘Yes’ under “Chronic disease module completed” in the corresponding row above. When all chronic disease modules have been completed continue on to Part6.*

**Part Three: Knowledge on Antibiotics and Resistance**

1. Have you ever heard of a type of medicine called an antibiotic?

1 Yes 0 No***🡪If no, skip to Q 3-4.***

1. Please mention the names of some antibiotics that you know. Wait for respondent to answer. *Tick "1"*if *medicine spontaneously mentioned. Write in name for medicines not on list. After that, probe for each name on the list, Tick "2" if agreed or “8” for ‘don't know’*

|  | **Spontaneous** | **After Probe** | **Don’t know** |
| --- | --- | --- | --- |
| 1. Amoxicillin | 1 | 2 | 8 |
| 1. Artemisinin and lumafantrine/ ALU | 1 | 2 | 8 |
| 1. Cotrimoxazole/Septrin | 1 | 2 | 8 |
| 1. Metronidazole/ Flagyl | 1 | 2 | 8 |
| 1. Mist Expectorant Sedative | 1 | 2 | 8 |
| 1. Paracetamol/Panadol | 1 | 2 | 8 |
| 1. Penicillin | 1 | 2 | 8 |
| 1. SP/ Fansidar | 1 | 2 | 8 |
| 1. Tetracycline | 1 | 2 | 8 |
| 1. Other (specify):………………………… | 1 |  |  |
| 1. Other (specify):………………………… | 1 |  |  |

1. For which of the following illnesses or conditions are antibiotics useful? *Read each illness or condition to respondent and tick “1” for Yes and “0” for No.*

|  | **Yes** | **No** |  |  | **Yes** | **No** |
| --- | --- | --- | --- | --- | --- | --- |
| Cough, cold and runny nose | 1 | 0 |  | Malaria | 1 | 0 |
| 1. Diarrhoea with bloody stool | 1 | 0 |  | Pneumonia | 1 | 0 |
| 1. Diarrhoea with watery stool | 1 | 0 |  | Sexually transmitted disease | 1 | 0 |
| 1. Pus discharging ear, earache | 1 | 0 |  | Tuberculosis | 1 | 0 |
| 1. HIV/AIDS | 1 | 0 |  |  |  |  |

1. Some medicines that used to work in the past for fighting infections are no longer working. This problem is called drug resistance. Have you ever heard of this problem?

| 1 Yes 2 No 3Don’t know |
| --- |
| **🡪 if Yes, continue below; otherwise go to Part 4: Acute Illness Module** |

1. Some illnesses now have serious problems with drug resistance because commonly used medicines no longer work for many patients. Can you tell me which of the following illnesses have more serious resistance problems? *Read each illness or condition to respondent and tick “1” for Yes and “0” for No.*

|  | **Yes** | **No** |  |  | **Yes** | **No** |
| --- | --- | --- | --- | --- | --- | --- |
| Cough, cold and runny nose | 1 | 0 |  | Malaria | 1 | 0 |
| 1. Diarrhoea with bloody stool | 1 | 0 |  | Pneumonia | 1 | 0 |
| 1. Diarrhoea with watery stool | 1 | 0 |  | Sexually transmitted disease | 1 | 0 |
| 1. Earache | 1 | 0 |  | Tuberculosis | 1 | 0 |
| 1. HIV/AIDS | 1 | 0 |  |  |  |  |

1. Can you tell me whether each of the following can contribute to causing resistance problems? *Read each illness or condition to respondent and tick “1” for Yes and “0” for No.*

| *Read statements, and tick one box for each statement* | **Yes** | **No** |
| --- | --- | --- |
| Stopping the medicine before all of the medicine is gone | 1 | 0 |
| 1. Taking the same medicine too frequently | 1 | 0 |
| 1. Taking more of the medicine at one time than is recommended | 1 | 0 |
| 1. Taking less of the medicine at one time than is recommended | 1 | 0 |
| 1. Skipping doses of the medicine | 1 | 0 |
| 1. Taking different medicines at the same time that interfere with each other | 1 | 0 |
| 1. Using medicines that were prescribed for someone else | 1 | 0 |
| 1. Using medicines that are poorly manufactured | 1 | 0 |
| 1. Taking medicines without eating any food | 1 | 0 |
| 1. Taking medicines that have been kept at home for a long time | 1 | 0 |
| 1. Any other reason? (specify): ………………………………………… | 1 | 0 |

1. **Part Four: Acute Illness Module**

*Fill one form for everyone in the household reported with an acute illness in Part Two (Question 2-5).* ***START WITH THE MOST RECENT SICK PERSON.***

| **Sick Person Name: ______________________** | **Roster Number\|__\|\|__\|** |
| --- | --- |

1. What type of health problems or symptoms did ***(first name)*** have during this illness? *Do not read. Tick one box for each group of symptoms mentioned:*

|  | **Yes** | **No** |  |  | **Yes** | **No** |
| --- | --- | --- | --- | --- | --- | --- |
| 1. Cold- cough, runny nose | 1 | 0 |  | 1. Bloody diarrhoea | 1 | 0 |
| 1. Tonsillitis- sore throat, painful swallowing | 1 | 0 |  | 1. Thirst, sweating | 1 | 0 |
| 1. Pneumonia-difficult or fast breathing | 1 | 0 |  | 1. Pain, aches | 1 | 0 |
| 1. Malaria-fever, headache, hot body | 1 | 0 |  | Bleeding, burn, accident | 1 | 0 |
| 1. Convulsions, fits | 1 | 0 |  | 1. Do not know | 1 | 0 |
| 1. STI/STD- genital discharge/ulcer | 1 | 0 |  | 1. Other (please specify): _____ | 1 | 0 |
| 1. Watery diarrhoea | 1 | 0 |  |  |  |  |

1. How serious do you think this illness was? *Read the choices. Tick one box.*

1 Very Serious 2 Somewhat Serious 3 Not Serious

1. At any point, did ***(first name)*** (or anybody else on his/her behalf) seek care for this illness outside the home?

1Yes 0No 🡪***if No, this module is complete Continue with the next person with acute illness. If there are no other acutely ill persons, go to Part Five: Chronic Illness Module.***

From which of the following sources of care did ***(first name)***receive care at any time during the illness?

*Read responses and tick one box for each of the categories:*

|  | **Yes** | **No** |  |  | **Yes** | **No** |
| --- | --- | --- | --- | --- | --- | --- |
| Public hospital | 1 | 0 |  | ADDO | 1 | 0 |
| 1. Mission or NGO hospital | 1 | 0 |  | Private pharmacy | 1 | 0 |
| 1. Public health center or dispensary | 1 | 0 |  | Drug store(DLBD) | 1 | 0 |
| 1. Private hospital or dispensary | 1 | 0 |  | Ordinary shop | 1 | 0 |
| 1. Traditional healer | 1 | 0 |  | Friend or neighbor | 1 | 0 |

*If an ADDO was one of the sources of care checked, complete the following two questions. Otherwise go to Q 4-7.*

Did you visit an ADDO as the **first** source of care for this illness 1Yes 0No

Did the ADDO attendant refer you to a public health care facility 1Yes 0No

1. Did (***first name***) take any medicine during the acute illness, including medicines taken during hospitalisation?

1 Yes 0No 🡪***if No, Go back to Q 4-1 (next person). If there is no other acutely ill persons go to Part Five: Chronic Illness Module***

1. Which medicines were taken during this illness?

*Write one medicine per row, and use codes provided in each column to collect information about each medicine.*

|  | **A** | **B** | **C** | | **D** | |
| --- | --- | --- | --- | --- | --- | --- |
|  | **Medicine** | **Route** | **Recommended / Prescribed by** | | **Obtained from** | |
|  | *Write name of medicine.*  *If name is not known, write the most detailed category given by respondent (“antibiotic’, “antimalaria”, “for fever”)* | *1 = oral*  *2 = injection*  *99 = other (specify)* | *1 = self*  *2 = household member*  *3 = friend/ neighbour*  *4 = doctor/nurse* | *5 = ADDO*  *6 = pharmacist*  *7 = drug seller*  *99 = other (specify)* | *1 = available at home*  *2 = friend or neighbour outside household*  *3 = public hospital*  *4 = NGO/mission hospital*  *5 = public health center* | *6 = private health care provider*  *7 = ADDO*  *8 =private pharmacy*  *9 = drug store (DLDB)*  *10 = ordinary shop*  *99 =other (specify)* |
| **Med 1** |  |  |  | |  | |
| **Med 2** |  |  |  | |  | |
| **Med 3** |  |  |  | |  | |
| **Med 4** |  |  |  | |  | |
| **Med 5** |  |  |  | |  | |
| **Med 6** |  |  |  | |  | |
| **Med7** |  |  |  | |  | |

1. How much did your household pay for medicines used to treat this illness? **|__||__||__||__||__||__|***Tshs*
2. Was any of this cost covered by health insurance? 2Yes, entirely 1Part of it was covered 0No
3. Did ***(first name)***take all of the medicines that were recommended or prescribed?

1 Yes 0No🡪***If No, continue with Q 4-12***

***If Yes, the acute module is now complete for this person – Continue with the next person with acute illness. If there is no other sick persons go to Part Five: Chronic Sick Module***

1. *If answer to Questions 4-11 is No, ask the following:* I am going to give you some possible reasons why ***(first name)*** did not take medicines as recommended. Which of these were reasons why?

| *Read statements, and tick one box for each statement* | **Yes** | **No** |
| --- | --- | --- |
| Symptoms have gotten better | 1 | 0 |
| 1. Someone in the household decided that medicines were not needed | 1 | 0 |
| 1. Someone advised not to take medicines | 1 | 0 |
| 1. Sick person had bad reactions to similar medicines in the past | 1 | 0 |
| 1. Someone in the household chose a different treatment | 1 | 0 |
| 1. The place where medicines can be obtained was too far away | 1 | 0 |
| 1. Medicines were not available at the public health care facility | 1 | 0 |
| 1. Medicines were not available at private pharmacy, drug store, or ordinary shop | 1 | 0 |
| 1. Medicines were not available at the ADDO | 1 | 0 |
| 1. Our household could not afford the medicines | 1 | 0 |
| 1. No one in the household could take time to obtain medicines | 1 | 0 |
| 1. Forgot to take the medicines | 1 | 0 |
| 1. Other (please specify): _________________ | 1 | 0 |

***The acute illness module is now complete for this person - continue with the next person with acute illness. If there are no other sick persons, go to Part Five: Chronic Illness Module***

**Part Five: Chronic Illness Module**

*Fill one form for everyone in the household reporting a chronic illness in Part Two.*

| **Sick Person Name: ______________________** | **Roster Number\|__\|\|__\|** |
| --- | --- |

1. Which of the following chronic diseases does ***(first name)***have? *Read responses in left column. Tick one box in the first group of responses for each disease. For each disease for which Yes is ticked, ask:* Has ***(first name)***been told by a doctor or other health care provider that he/she should be taking medicines to treat this disease?

|  | **A: Has this disease?** | | | **B: Told to take medicines for this disease?** | | |
| --- | --- | --- | --- | --- | --- | --- |
|  | **Yes** | **No** | **Do not Know** | **Yes** | **No** | **Do not Know** |
| Hypertension, high blood pressure | 1 | 0 | 99 | 1 | 0 | 99 |
| 1. Heart disease, heart attack consequence | 1 | 0 | 99 | 1 | 0 | 99 |
| 1. Diabetes, high blood sugar | 1 | 0 | 99 | 1 | 0 | 99 |
| 1. Asthma, wheezing, chronic difficulty breathing | 1 | 0 | 99 | 1 | 0 | 99 |
| 1. HIV infection, AIDS | 1 | 0 | 99 | 1 | 0 | 99 |
| 1. Arthritis, chronic body pain | 1 | 0 | 99 | 1 | 0 | 99 |
| 1. Epilepsy, seizures, fits | 1 | 0 | 99 | 1 | 0 | 99 |
| Ulcer, chronic stomach pain | 1 | 0 | 99 | 1 | 0 | 99 |
| Stroke consequence | 1 | 0 | 99 | 1 | 0 | 99 |
| High cholesterol | 1 | 0 | 99 | 1 | 0 | 99 |
| Cancer | 1 | 0 | 99 | 1 | 0 | 99 |
| Tuberculosis | 1 | 0 | 99 | 1 | 0 | 99 |
| Liver disease | 1 | 0 | 99 | 1 | 0 | 99 |
| Depression | 1 | 0 | 99 | 1 | 0 | 99 |
| Other (please specify): | 1 | 0 | 99 | 1 | 0 | 99 |

🡪***If no chronic diseases were checked or no medicines were recommended, this chronic module is complete – continue with the next person with chronic illness.***

1. Which medicines has ***(first name)***been told to take **regularly** for these chronic diseases or another chronic condition? *Write one medicine per row, using codes in the previous table to identify the condition.*

|  | **A** | **B** | | **C** | **D** | | **E** | **F** | |
| --- | --- | --- | --- | --- | --- | --- | --- | --- | --- |
|  | **Medicine** | **Recommended/Prescribed by** | | **Condition for which recommended** | **Number of days’ supply obtained last month** | | **Cost for this medicine last month** | **Any cost paid by insurance last month?** | |
|  | *Write name of medicine. If name is not known, write the category given by respondent (“antibiotic”, “for depression”, “for diabetes”)* | *1=Doctor/ nurse*  *2=Community health worker*  *3= Pharmacy/chemist*  *4=ADDO worker*  *5=Drug store worker (DLDB)* | *6=Friend/Neighbor*  *7=Relative*  *8=Respondent him/herself*  *96=Other (specify))*  *98=Don’t know* | *Write code from previous question (i.e. “a”. for hypertension, etc.)Write “X” if unknown* | *In days*  *Write “0” if no medicine was obtained last month.* | | *In Tshs*  *Write “0” if not obtained last month or if obtained free.* | *Tick Yes if insurance covered some of the cost.*  *Tick No otherwise.* | |
| **Med 1** |  |  | |  |  | | **\|__\|\|__\|\|__\|\|__\|\|__\|\|__\|** | 1 Yes | 0 No |
| **Med 2** |  |  | |  |  | | **\|__\|\|__\|\|__\|\|__\|\|__\|\|__\|** | 1 Yes | 0 No |
| **Med 3** |  |  | |  |  | | **\|__\|\|__\|\|__\|\|__\|\|__\|\|__\|** | 1 Yes | 0 No |
| **Med 4** |  |  | |  |  | | **\|__\|\|__\|\|__\|\|__\|\|__\|\|__\|** | 1 Yes | 0 No |
| **Med 5** |  |  | |  |  | | **\|__\|\|__\|\|__\|\|__\|\|__\|\|__\|** | 1 Yes | 0 No |
| **Med 6** |  |  | |  |  | | **\|__\|\|__\|\|__\|\|__\|\|__\|\|__\|** | 1 Yes | 0 No |
| **Med 7** |  |  | |  |  | | **\|__\|\|__\|\|__\|\|__\|\|__\|\|__\|** | 1 Yes | 0 No |
| **Med 8** |  |  | |  |  | | **\|__\|\|__\|\|__\|\|__\|\|__\|\|__\|** | 1 Yes | 0 No |
| **Med 9** |  |  | |  |  | | **\|__\|\|__\|\|__\|\|__\|\|__\|\|__\|** | 1 Yes | 0 No |
| **Med 10** |  |  | |  |  | | **\|__\|\|__\|\|__\|\|__\|\|__\|\|__\|** | 1 Yes | 0 No |
| **All Medicines** | | *Ask for the total cost of all medicines obtained last month only if the cost of each individual medicine was not known* | | | |  | **\|__\|\|__\|\|__\|\|__\|\|__\|\|__\|** | Tshs. |  |

1. Sometimes people cannot take all medicines as recommended. Does ***(first name)*** usually take all of his/her medicines as recommended?

1 Yes *🡪****if Yes, the chronic module is now complete for this person –*** 0No

***continue with the next person with a chronic illness***

1. *If answer to Question 5-3is No, ask the following question:* I am going to read some possible reasons why ***(first name)*** may not always take medicines as recommended. Can you tell me whether each of these is a reason why he/she sometimes does not take the recommended medicines?

| *Read statements, and tick one box for each statement.* | **Yes** | **No** |
| --- | --- | --- |
| 1. Symptoms have gotten better | 1 | 0 |
| 1. Someone in the household decided medicines were not needed | 1 | 0 |
| 1. Someone advised not to take medicines | 1 | 0 |
| 1. Sick person had bad reactions to medicines in the past | 1 | 0 |
| 1. Someone in the household chose a different treatment | 1 | 0 |
| 1. The place where medicines can be obtained is too far away | 1 | 0 |
| 1. Medicines are not available at the public health care facility | 1 | 0 |
| 1. Medicines are not available at private pharmacy, drug store or ordinary shop | 1 | 0 |
| 1. Medicines are not available at ADDO | 1 | 0 |
| 1. Our household cannot afford the medicines | 1 | 0 |
| 1. No one in the household can take time to obtain medicines | 1 | 0 |
| 1. Forgot to take the medicines | 1 | 0 |
| 1. Other (please specify): _________________ | 1 | 0 |

🡪***This chronic module is now complete for this person - continue with the next person with a chronic illness.***

**Part Six: Household Medicines**

1. Do you have any medicines available at home today?

1 Yes 0No 🡪**if No, Skip to Question 7-1**

1. Can I please see all of them? *Write one medicine per row, and use codes provided in each column to collect information about each medicine.*

|  | **A** | **B** | | **C** | **D** | **E** | | **F** | | **G** | |
| --- | --- | --- | --- | --- | --- | --- | --- | --- | --- | --- | --- |
|  | **Medicine** | **Recommended/Prescribed by** | | **Obtained from** | **In home because** | ***Using medicine*** | | **Label OK** | | **Primary Package OK** | |
|  | *Write name of medicine.*  *If name is not known, write the category given by respondent (“antibiotic”, “antimalaria”, “for fever”)* | *1=Doctor/ nurse*  *2=Community health worker*  *3=Pharmacist/chemist*  *4=ADDO worker*  *5=Drug store worker (DLDB)* | *6=friend/neighbour*  *7=relative*  *8=respondent her/himself*  *99=others (specify)*  *98= don’t know* | *1= family, friend*  *2= public hospital*  *3= NGO/mission hospital*  *4= public health center/ dispensary*  *5= private provider*  *6= private pharmacy*  *7= ADDO*  *8= drug store (DLDB)*  *9=ordinary shop*  *99=other (specify)* | *1=current treatment*  *2=left from past treatment*  *3=anticipate future need* | *Today or yesterday, did anyone in the household take any of these medicines?* | | *Tick Yes if label includes medicine name, dose, and expiration date.*  *Otherwise tick No* | | *Tick Yes if primary package is an envelope or a closable container, and if it contains only one medicine.*  *Otherwise tick No* | |
| **Med 1** |  |  | |  |  | 1Yes | 0 No | 1Yes | 0 No | 1Yes | 0 No |
| **Med 2** |  |  | |  |  | 1Yes | 0 No | 1Yes | 0 No | 1Yes | 0 No |
| **Med 3** |  |  | |  |  | 1Yes | 0 No | 1Yes | 0 No | 1Yes | 0 No |
| **Med 4** |  |  | |  |  | 1Yes | 0 No | 1Yes | 0 No | 1Yes | 0 No |
| **Med 5** |  |  | |  |  | 1Yes | 0 No | 1Yes | 0 No | 1Yes | 0 No |
| **Med 6** |  |  | |  |  | 1Yes | 0 No | 1Yes | 0 No | 1Yes | 0 No |
| **Med 7** |  |  | |  |  | 1Yes | 0 No | 1Yes | 0 No | 1Yes | 0 No |
| **Med 8** |  |  | |  |  | 1Yes | 0 No | 1Yes | 0 No | 1Yes | 0 No |
| **Med 9** |  |  | |  |  | 1Yes | 0 No | 1Yes | 0 No | 1Yes | 0 No |
| **Med 10** |  |  | |  |  | 1Yes | 0 No | 1Yes | 0 No | 1Yes | 0 No |
| **Med 11** |  |  | |  |  | 1Yes | 0 No | 1Yes | 0 No | 1Yes | 0 No |
| **Med 12** |  |  | |  |  | 1Yes | 0 No | 1Yes | 0 No | 1Yes | 0 No |

**Part Seven: Opinions about Medicines and ADDOs**

I am now going to read you some opinions about health care, medicines, and ADDOs. There are no correct answers. For each opinion, please tell me whether you agree or disagree. *Do not read the option “Do not know”. Tick it if the respondent is unable to choose “agree” and “disagree”. Tick only one box for each statement.*

1. First, I am going to ask some opinions about access to care and medicines. **Agree Disagree Don’t know**

| 1. Different names may be used for the same medicine. | 1 | 2 | 3 |
| --- | --- | --- | --- |
| 1. ADDOs are allowed to sell different types of medicines than drug stores. | 1 | 2 | 3 |
| 1. You can always obtain antibiotics at the ADDO when you need them. | 1 | 2 | 3 |
| 1. The public health care facility where you usually seek care has the medicines you need. | 1 | 2 | 3 |
| 1. The waiting time at the public health care facility where you usually seek care is reasonable. | 1 | 2 | 3 |
| 1. The ADDO closest to your household usuallyhas the medicines you need. | 1 | 2 | 3 |
| 1. ADDOs are the most convenient places to seek care in your community. | 1 | 2 | 3 |
| 1. ADDO staff sometimes refer customers to public health facilities for care. | 1 | 2 | 3 |

1. Now I am going to ask some opinions about cost of medicines. **Agree Disagree Don’t know**

| 1. Your household can get free medicines at the public health care facility. | 1 | 2 | 3 |
| --- | --- | --- | --- |
| 1. Medicines are more expensive at ADDOs than in the public health care facility. | 1 | 2 | 3 |
| 1. It is easy for you to find out how much medicines cost in different outlets in your community. | 1 | 2 | 3 |
| 1. When you receive a prescription from a doctor or health worker, you are comfortable asking how much the medicines will cost. | 1 | 2 | 3 |
| 1. Two identical medicines may be sold at different prices. | 1 | 2 | 3 |
| 1. You know where to find medicines at the lowest price in your community. | 1 | 2 | 3 |
| 1. When you buy a medicine, you normally ask if there is a less expensive product. | 1 | 2 | 3 |
| 1. Your household can usually afford to buy the medicines you need. | 1 | 2 | 3 |
| 1. In the past, your household has had to borrow money or sell things to pay for medicines. | 1 | 2 | 3 |
| 1. Your household would be more likely to obtain medicines at an ADDO if insurance reimbursed their cost. | 1 | 2 | 3 |
| 1. In the ADDO, the counter attendant takes into account your ability to pay when they decide which medicines to sell. | 1 | 2 | 3 |
| 1. Your household can usually get credit from the ADDO if you need it. | 1 | 2 | 3 |
| 1. In the public health care facility where you usually seek care, they take into account your ability to pay when deciding which medicines to prescribe. | 1 | 2 | 3 |
| 1. When the counter attendant in an ADDO recommends a medicine, you can be sure that it is the best value for money. | 1 | 2 | 3 |

1. Now I am going to ask your opinions about quality of services and medicines. **Agree Disagree Don’t know**

| 1. The quality of services is good at ADDOs in your community. | 1 | 2 | 3 |
| --- | --- | --- | --- |
| 1. The quality of services is good at the public health care facility where you usually seek care. | 1 | 2 | 3 |
| 1. The quality of services is better in ADDOs than in drug stores | 1 | 2 | 3 |
| 1. You trust the ADDO counter attendant to give the right advice about treatment. | 1 | 2 | 3 |
| 1. When the counter attendant in an ADDO recommends a medicine, you can be sure that it is of good quality. | 1 | 2 | 3 |
| 1. Medicines of better quality are more expensive. | 1 | 2 | 3 |
| 1. There are places in your community where you would never buy medicines because they sell medicines of poor quality. | 1 | 2 | 3 |

1. The last set of opinions is about antibiotics. **Agree Disagree Don’t know**

| 1. Antibiotics such as tetracycline and ampicillin are not useful in treating a cold. | 1 | 2 | 3 |
| --- | --- | --- | --- |
| 1. Antibiotics such as tetracycline and ampicillin are useful in treating watery diarrhoea. | 1 | 2 | 3 |
| 1. Some antibiotics that used to work in the past for fighting infections are no longer working. | 1 | 2 | 3 |
| 1. Some antibiotics fail to work when one stops taking them before completing the full course. | 1 | 2 | 3 |
| 1. Using antibiotics prescribed for someone else can be harmful. | 1 | 2 | 3 |

**Part Eight: Assets and Medicines Expenditures**

Finally I would like to ask a few questions about the possessions in your home and how much your household spends.

1. Can you please tell me how many rooms including bedrooms, dining room, kitchen that are in your home?**|__| |__|**
2. Does anyone in your household has a:

|  | **Yes** | **No** |  |  | **Yes** | **No** |  |  | **Yes** | **No** |
| --- | --- | --- | --- | --- | --- | --- | --- | --- | --- | --- |
| 1. Watch | 1 | 0 |  | 1. Motorcycle | 1 | 0 |  | 1. Power tiller | 1 | 0 |
| 1. Bicycle | 1 | 0 |  | 1. Car or truck | 1 | 0 |  | 1. Mobile phone | 1 | 0 |
| 1. Radio | 1 | 0 |  | 1. Tractor | 1 | 0 |  | 1. Land | 1 | 0 |

1. Does your household have:

| 1. Tap/running water inside house: | | | 1Yes | 0No |  | 1. Electric power: | 1Yes | 0No |
| --- | --- | --- | --- | --- | --- | --- | --- | --- |
| 1. Toilet: | 1Yes, its own | 2Yes, shared | | 0 No |  | **🡪If no electric power, Skip to Question 8-5** | | |

1. Does anyone in your household have:

|  | **Yes** | **No** |  |  | **Yes** | **No** |  |  | **Yes** | **No** |
| --- | --- | --- | --- | --- | --- | --- | --- | --- | --- | --- |
| 1. Television | 1 | 0 |  | 1. Refrigerator | 1 | 0 |  | 1. Electric Kettle | 1 | 0 |
| 1. Electric Iron | 1 | 0 |  | 1. Electric Cooker | 1 | 0 |  | 1. Microwave | 1 | 0 |

1. I will now give you five different levels of spending. Please choose the level that is closest to what your household spent in total over thepast 4 weeks. *(Read ranges of expenditures corresponding to the size of this household from the income table.)*

1A 2B 3C 4D 5E

1. Can you provide the actual total amount? 1Yes, **|__||__||__||__||__||__|** *inTSh*0No

***Thank the respondent and reassure him/her about the confidentiality of answers.***
